# Stakeholder Participation for Legitimate Priority Setting: A Checklist

**DOI:** 10.15171/ijhpm.2018.57

**Published:** 2018-07-04

**Authors:** Maarten P.M. Jansen, Rob Baltussen, Kristine Bærøe

**Affiliations:** ^1^Department for Health Evidence, Radboud Institute for Health Sciences, Radboud University Medical Center, Nijmegen, The Netherlands.; ^2^Department of Global Public Health and Primary Care, University of Bergen, Bergen, Norway.

**Keywords:** Priority Setting, Accountability for Reasonableness, Legitimacy, Stakeholder Participation

## Abstract

Accountable decision-makers are required to legitimize their priority setting decisions in health to members of society. In this perspective we stress the point that fair, legitimate processes should reflect efforts of authorities to treat all stakeholders as moral equals in terms of providing all people with well-justified, reasonable reasons to endorse the decisions. We argue there is a special moral concern for being accountable to those who are potentially adversely affected by decisions. Health authorities need to operationalize this requirement into real world action. In this perspective, we operationalize five key steps in doing so, in terms of (*i*) proactively identifying potentially adversely affected stakeholders; (*ii*) comprehensively including them in the decision-making process; (*iii*) ensuring meaningful participation; (*iv*) communication of recommendations or decisions; and (*v*) the organization of evaluation and appeal mechanisms. Health authorities are advised to use a checklist in the form of 29 reflective questions, aligned with these five key steps, to assist them in the practical organization of legitimate priority setting in healthcare.

## Introduction

Health authorities make priority setting decisions on behalf of society. Their decisions are bound to be controversial as stakeholders likely disagree over which priorities should be set and over who should benefit and who should not. Increasingly, there is attention for organizing ‘fair, legitimate decision-making processes’ and organizing ‘public deliberation.’ In line with this trend, health authorities often organize some form of stakeholder participation, embedded in their decision-making processes.^1^ In doing so they may use different deliberative methods, identified and described elsewhere.^1,2^ Reasons for organizing stakeholder participation can be multiple, eg, gaining an understanding of the stakeholders’ values and how a specific decision affects these values, enhancing the epistemic outcome of deliberation, educating the public, or promoting democracy by making citizens co-decision-makers.^1-4^

Whatever the health authorities’ specific reason for organizing stakeholder participation, fair, legitimate processes have to reflect efforts of authorities to treat all stakeholders as moral equals and thus provide people with well-justified, reasonable reasons to endorse the process and thus the decision – even if it is the case that they would have preferred another outcome.^5,6^ This is especially relevant for stakeholders who carry the negative consequences of a decision, the so-called adversely affected stakeholders.

In practice, health authorities lack easy-to-use tools that can support them in carefully organizing meaningful stakeholder participation. As such, there is a risk that participation of stakeholders, if organized at all, merely reflects tokenism rather than justified and adequately integrated participation. In response to this, we here present a checklist for carefully organizing stakeholder participation. This can help health authorities in being accountable for their priority setting decisions, especially towards adversely affected stakeholders.

## Why Are Health Authorities Accountable to (Adversely Affected) Stakeholders?

Priority setting in health is recognized as a value-laden political process, which takes place in an environment of diverging social values and interests.^7-13^ Indeed, members of society, or stakeholders, may carry a wide range and diversity of social values, such as maximizing population health, doing no harm, avoiding catastrophic expenditure, or giving priority to the worse off.^14-16^ According to liberal theory, stakeholders may *reasonably* disagree on the relative importance of such values and as well on how some of them are specified during priority setting.^17^

Health authorities make priority setting decisions on behalf of society, eg, when deciding on the public reimbursement of new health interventions. The power that health authorities possess to make priority setting decisions on behalf of societies, characterized by representing this value pluralism, is justified only in so far decision-making is carried out in *legitimate* ways.

Legitimate decision-making requires that processes reflect efforts of authorities to treat all stakeholders as moral equals.^5^ Ideally, this means all stakeholders would have good reasons to endorse the decision-making process as fair - even those who would have preferred another outcome than the resulting decision.^5,6^ In order to enjoy the moral authority to make priority setting decisions in health (as opposed to some contingent social power), there is an ethical demand on health authorities to be accountable to adversely affected stakeholders.^5^ Independently of what kind of normative theory one endorses, it can be seen as a fundamental ethical requirement, that those who are carrying the burdens of the decisions are (*i*) explicitly recognized as being stakeholders; and (*ii*) entitled to being provided with good reasons to appreciate the decision-making process as fair. If these concerns are not taken seriously by decision-makers they lose their moral authority for making priority setting decisions in health. Subsequently, they may undermine the legitimacy of their own decision-making process.^18^

## Who Are the Adversely Affected Stakeholders?

We distinguish between four reasons why stakeholders can be considered adversely affected by decisions: (*i*) stakeholders experience a health loss as a direct result of a priority setting decision (*the health loss reason)*; (*ii*) stakeholders experience a health loss as an indirect result of a priority decision, which is the case when a newly approved health intervention displaces their personally needed intervention (*the indirect health loss reason)*; (*iii*) stakeholders need to communicate decisions which may adversely affect the patient-clinician relationship (*the communication reason*)^19^; (*iv*) stakeholders are responsible for implementing a decision they strongly disagree with (*the integrity reason)*. This list is not necessarily exhaustive: further categories may be added according to a specific burden inflicted by a decision.

## Development of the Checklist

Important academic work on the conditions of fair processes is the accountability for reasonableness (A4R) framework.^10,20^ This framework identifies four key conditions for organizing fair processes: (*i*) organizing deliberation among stakeholders to identify relevant rationales; (*ii*) ensuring transparency of the decisions; (*iii*) organization of appeal opportunities; and (*iv*) regulation of conditions i-iii.^10,20^ However, there is a gap in the literature on how to comprehensively translate these requirements into practice in general,^21^ and more specifically when it comes to ensuring accountability towards the adversely affected identified above.^5^

We developed a checklist by operationalizing the A4R framework and reflecting on the ethical notions it invokes, supported by broader literature on stakeholder participation and public deliberation, paying special concern for the ethical demand to be accountable to adversely affected stakeholders.^2-5,20,22-27^ Based on this reflection we defined five key actionable steps in being accountable to adversely affected stakeholders: (*i*) proactively identifying potentially adversely affected stakeholders; (*ii*) comprehensively including stakeholders; (*iii*) ensuring meaningful participation; (*iv*) communication of recommendations and/or decisions; and (*v*) the organization of evaluation and appeal mechanisms – further explained below. In doing so, we did not assume a generic model of decision-making, as real-world decision-making processes are unlikely to take place in a chronological and tidy manner. Further reflection on what it would entail to actually operationalize these five steps was carried out in accordance with the underlying imperative of treating people as moral equals. A simple test of whether the inclusion of stakeholders meets this imperative or not, is to imagine whether we would have felt respectfully recognized and properly involved on the suggested terms ourselves. By following this methodology we could spell out questions that health authorities should think about when they organize stakeholder participation. This reflection process resulted in a shortlist of questions for use by health authorities. The five steps and their respective questions are together presented as a reflective checklist (Box 1). The checklist is not meant to be all-encompassing or exhaustive, rather, it is meant to cover key concerns and invoke reflection by health authorities on the most relevant and actionable choices they make. Therefore, the checklist should be taken as a starting point for discussion and future adjustment.

**Box 1. A Checklist for Stakeholder Participation**

** A. Identification of potentially adversely affected stakeholders**

1. Who may experience a health loss as a result of a negative decision?
2. Who may experience a health loss as a result of a positive decision?
3. Who may be adversely affected because they are responsible for communicating the decision?
4. Who may be adversely affected because they are responsible for implementing the decision?

**
B. Comprehensive stakeholder inclusion
**

1. Are all relevant stakeholders informed about the possibility and procedures of participation?
2. Is participation organized in a way that effectively and efficiently facilitates the inclusion of stakeholders?
3. Are efforts made to include all relevant, especially difficult-to-reach, stakeholders?
4. Can stakeholders participate in the identification and topic selection of health services for evaluation?
5. Can stakeholders participate in the scoping of relevant questions for evaluation?
6. Can stakeholders participate in the development of recommendations?
7. Can stakeholders participate in the evaluation of decisions?
8. Are alternative non-participatory strategies used for inclusion of stakeholders’ values?

**
C. Meaningful stakeholder participation
**

1. Are stakeholders fully and in time informed about the available evidence?
2. Is argumentation and evidence presented in a way that is understandable to all relevant stakeholders?
3. Can stakeholders freely voice their perspectives (ie, no stakeholder is allowed to dominate a discussion or activity)?
4. Are stakeholder perspectives addressed in respectful and courteous ways?
5. Do stakeholders have sufficient time to provide input?
6. Are stakeholder perspectives equally accounted for in the deliberation?
7. Is it clear to all stakeholders involved how their input is going to be considered, scrutinized and put to use?
8. Can stakeholders actively interact in the deliberation?
9. Is further evidence collection considered when judged relevant and feasible?

**
D. Transparent communication of recommendations and/or decisions
**

1. Is information provided on the underlying argumentation and process to come to a recommendation and/or decision?
2. Is input from stakeholders documented and addressed explicitly?
3. Are recommendations and/or decisions clearly communicated?
4. Are stakeholders informed in time on the recommendation and/or decision?

**
E. Appeal and evaluation
**

1. Can stakeholders easily make an appeal on the underlying argumentation or process? Are appeals documented and publicly accessible?
2. Are appeals handled consistently and is justification provided in an understandable way?
3. Are mechanisms in place to revise decisions or the process based on appeals?

### Step 1: Identifying Potentially Adversely Affected Stakeholders

Authorities must make efforts to systematically identify potentially adversely affected stakeholders before making a decision, as to ensure that stakeholders’ perspectives, suggestions and arguments will enter into the decision-making process, preferably as early as possible during the deliberative process.^5^ The first step is to identify real world persons as representatives of the potentially adversely affected stakeholders according to the categories we identified above.

### Step 2: Including Stakeholders in the Decision-Making Process

Identified stakeholders must be included in the decision-making process.^4,5,22^ This demands a pro-active attitude of health authorities, which starts with inviting stakeholders to attend meetings and ensuring meetings are accessible.^22-24^ Specifically, efforts should be made to ensure that known hard-to-reach stakeholders actually have reasonable opportunities to participate.^24^ Alternatively, strategies for including stakeholders values, other than their direct participation, should be explored pro-actively to ensure the uptake of arguments – in line with broader consultation and communication efforts as defined elsewhere.^2,5^

### Step 3: Ensuring Meaningful Participation

Efforts must be made to ensure meaningful participation. This requires that stakeholders can actively interact in the deliberation, freely voice their perspectives and that they are treated with due respect – while being provided with sufficient time to do so.^3,5,11,22,23,25^ Also, it requires that further evidence is considered or commissioned when this is feasible. Furthermore, all evidence and argumentation put forward should be presented to stakeholders in time and carefully addressed in a way that is understandable to all stakeholders.^5,22,25^ Importantly, this requires that their input is considered, put to use, scrutinized and not ignored – and that its clear at the outset of a process, to all stakeholders involved, how divergent views and interests are to be resolved and this concern is to be satisfied.^5^ Overall, the deliberative process should provide opportunities for mutual learning.^3,5,23,26,27^

### Step 4: Communicating Recommendations and/or Decisions in Understandable and Transparent Ways

The final recommendations and/or decisions have to be determined by acceptable standards of voting or left to the discretion of legitimately appointed decision-makers. Decisions should be communicated shortly after being made, in transparent ways that help shape understanding among stakeholders of why the specific decision was made.^4,5^ In doing so, health authorities should provide clear argumentation for why those who become adversely affected by their decision are to carry the burden.^5^

### Step 5: Organizing Evaluation and Appeal Options

User-friendly evaluation and appeal options should be organized.^4,10^ Any appeals made should be documented and made anonymous and publicly available. Moreover, mechanisms should be put in place that ensure all appeals are handled with care – providing justification of the appeals outcome by decision-makers in an understandable way.^5^

## How to Use the Checklist?

Health authorities can use the checklist to revise for possible shortcomings of current processes and install mechanisms for improvement. As mentioned, the checklist is generic in nature and questions included in the checklist are relevant to reflect on throughout a decision-making process. In practice, processes may be split-up into specific steps (eg, assessment and appraisal) for which certain questions may be more (or less) relevant to consider. Also, if decision-making is split-up into separate steps, it may well be that each step requires a different answer to the same question. Furthermore, answers to questions are context-specific and there is no decisive evidence on what would constitute ‘right answers’ to these individual questions. In some contexts it may eg, be reasonable to reimburse travel expenses for the sake of accessibility, while in other cases this may be judged irrelevant or inappropriate. Nevertheless, health authorities are advised to inform their specific choices by evidence if available – or to learn from other countries’ experiences. Finally, the checklist reflects an aspirational goal of ideal accountability of decision-makers to adversely affected stakeholders. Authorities should take incremental steps towards meeting this goal by prioritizing specific efforts according to local needs and affordances.

## Conclusion

Accountable decision-makers are required to legitimize their priority setting decisions in health to members of society. Health authorities need to operationalize this requirement into real world action. In this perspective, we have argued for five key steps in doing so, in terms of proactively identifying potentially adversely affected stakeholders, comprehensively including them in the decision-making process, ensuring meaningful participation, communication of recommendations or decisions, and the organization of evaluation and appeal mechanisms. Health authorities are advised to use the provided checklist in the form of 29 reflective questions to assist them in the practical organization of legitimate priority setting in healthcare.

## Acknowledgements

We would like to acknowledge Antonio Ciaglia (policy manager at the International Alliance of Patients’ Organizations), Gert Jan van der Wilt (Professor of Health Technology Assessment at the Department for Health Evidence, Radboud University Medical Center, Nijmegen, the Netherlands) and Matthew McCoy (Assistant Professor of Medical Ethics and Health Policy at the Hospital of the University of Pennsylvania, Philadelphia, PA, USA) for their comments on the first draft of the paper. In addition, the work was presented at the Priorities 2016 conference in Birmingham.

## Ethical issues

Not applicable.

## Competing interests

Authors declare that they have no competing interests.

## Authors’ contributions

KB had the idea for this paper. All authors contributed to the outline of the paper, MPMJ wrote the first draft of this paper, after which RB and KB provided suggestions and comments throughout the process of drafting the final version of the manuscript.

## Authors’ affiliations

^1^Department for Health Evidence, Radboud Institute for Health Sciences, Radboud University Medical Center, Nijmegen, The Netherlands. ^2^Department of Global Public Health and Primary Care, University of Bergen, Bergen, Norway.

## Funding

MPMJ and RB are funded through the Netherlands Organization for Scientific Research (NWO) Talent Scheme Vici. KB’s postdoc position is funded by the University of Bergen, Bergen, Norway.
